# Abstract Action Language Processing in Eleven-Year-Old Children: Influence of Upper Limb Movement on Sentence Comprehension

**DOI:** 10.3390/bs11120162

**Published:** 2021-11-23

**Authors:** Larissa S. Balduin-Philipps, Sabine Weiss, Franziska Schaller, Horst M. Müller

**Affiliations:** 1 Experimental Neurolinguistics Group, Department of Linguistics, Bielefeld University, Universitätsstrasse 25, 33615 Bielefeld, Germany; sabine.weiss@uni-bielefeld.de (S.W.); franziska.schaller@gmail.com (F.S.); horst.mueller@uni-bielefeld.de (H.M.M.); 2 Center for Cognitive Interaction Technology (CITEC), Bielefeld University, Inspiration 1, 33619 Bielefeld, Germany; 3 Clinical Linguistics, Department of Linguistics, Bielefeld University, Universitätsstrasse 25, 33619 Bielefeld, Germany

**Keywords:** embodiment, children, abstract language processing, action verb, concrete language

## Abstract

Regarding the embodiment of language processing in adults, there is evidence of a close connection between sensorimotor brain areas and brain areas relevant to the processing of action verbs. This thesis is hotly debated and has therefore been thoroughly studied in adults. However, there are still questions concerning its development in children. The present study deals with the processing of action verbs in concrete and abstract sentences in 60 eleven-year-olds using a decision time paradigm. Sixty-five children mirrored arm movements or sat still and rated the semantic plausibility of sentences. The data of the current study suggest that eleven-year-olds are likely to misunderstand the meaning of action verbs in abstract contexts. Their decision times were faster and their error rates for action verbs in concrete sentences were lower. However, the gender of the children had a significant influence on the decision time and the number of errors, especially when processing abstract sentences. Females were more likely to benefit from an arm movement before the decision, while males were better if they sat still beforehand. Overall, children made quite a few errors when assessing the plausibility of sentences, but the female participants more often gave plausibility assessments that deviated from our expectations, especially when processing abstract sentences. It can be assumed that the embodiment of language processing plays some role in 11-year-old children, but is not yet as mature as it is in adults. Especially with regard to the processing of abstract language, the embodied system still has to change and mature in the course of child development.

## 1. Introduction

With respect to the way language is processed, there are two opposing viewpoints in cognitive linguistics: On the one side, strongly amodal theories describe language and cognition as two separate systems. In this case, language processing relies on abstract, amodal symbols that are completely detached from sensory simulation [1,2,3]. Traces of sensory perception are stored as such and translated into an abstract symbol that is bound to the original sensory stimulation by an arbitrary connection. On the other side, modal cognition theories propose that meaning is represented in a widespread and multimodal cognitive network in which language processing is related to or even is sensorimotor simulation [4,5]. This modal aspect is expressed in various, differently strong embodied theories of language. In most of these theories, mental simulation—described as the reactivation of neuronal traces resulting from interactions with the environment and the body—plays an essential role.

With respect to action verb processing, mental simulations of motor experience are especially important. Motor representations are action plans that can be reactivated to achieve certain goals when interacting with the environment [6]. The difference between the simulation of a mental motor representation and an actual action is a weaker activation of motor areas, the suppression of motor output, and a lack of sensory feedback from an actual muscle movement [6]. In addition, it has been shown that the brain systems for motor imagery (and probably mental motor simulation), action observation, and the actual movement execution only partly overlap anatomically [7,8].

Some studies examined the role primary or secondary motor areas might play in simulating information during action verb processing using action priming [9,10,11]. Glenberg and Kaschak [10] described the Action-Sentence Compatibility Effect (ACE), which suggests a pre-activation of motor-related cortical areas due to action language processing. In Glenberg and Kaschak’s [12] study, participants were asked to make a semantic decision by pressing a button after processing sentences containing action information. Congruence between the movement executed when pressing the button and the movement described in the sentence—in both cases, movements towards or away from the body—led to shorter reaction times, while incongruence led to longer reaction times. Therefore, Glenberg and Kaschak [12] suggested that the activation of similar neural structures is requested for action language comprehension and movement execution.

A facilitation effect induced by motor priming is also reported by Wilson and Gibbs [13]. In their experimental condition, participants performed previously learned body movements. After each movement, they read metaphorical sentences containing action verbs and subsequently pressed a button to indicate sentence comprehension. Decision times for stimuli in which the body movement and the action verb matched were shorter compared to stimuli in which the performed movement and read action verb mismatched [13]. It is important to note that the activation of neural structures for action language comprehension could also lead to an interference effect rather than a facilitation (priming) effect. One reason for this might be the presentation of stimuli recruiting the same neural structures in quick succession [14,15].

In abstract language processing, the ACE is also observable [10]. According to the authors, this speaks in favour of a motor priming effect in connection with abstract language processing. However, the degree to which abstract language is embodied and can be influenced by motor priming is highly debated. fMRI studies argue in favour of a gradual activation of motor areas in action verb processing because the activation of sensorimotor areas decreases with an increasing level of abstraction [16,17]. These findings on adults are in line with the results of a behavioural study by Schaller et al. [18] and an EEG-study by Schaller et al. [19] who analysed sensorimotor activation in a decision time paradigm. Participants assessed the plausibility of sentences containing action verbs in concrete and abstract contexts after mirroring movements presented in videos. While only congruent movements led to facilitation in a concrete context, both congruent and incongruent movements eased abstract action verb processing. These results speak in favour of a gradual involvement of motor areas and a decrease in movement specificity in processing action verbs in an abstract context.

In children, competence in abstract language comprehension only seems to develop in the course of adolescence [20]. Theories of language development differ greatly with regard to the proposed age of complete language acquisition and postulate it to be between the twelfth [21] and twentieth year of age [22]. Due to weaker mental representations, e.g., for idioms, the comprehension of abstract content is not fully developed by the age of twelve [23]. For example, a lack of pragmatic language skills could be observed up to adolescence [24]. As a result, it is to be expected that children, compared to adults, have more problems processing verbs in abstract than in concrete contexts, and therefore make comparatively more errors and are slower in making task-related decisions [25].

These steps in language development correspond closely with the development of language-relevant brain regions at frontal and temporal regions, which show maturation processes well into adulthood [26]. Such long maturation processes give rise to the questions of whether the relationship between the sensorimotor system and language postulated in embodiment theory function in the same way and if it is based on the same mechanisms in children as in adults. Such a similarly functioning relationship could be more easily assumed for the processing of concrete language, but the question remains of whether children of this age also process abstract language on the basis of sensorimotor simulations, as adults often do.

Embodiment in language development has been studied to a lesser extent in children than in adults. It is undisputed that infants and young children first use sensorimotor knowledge and interactions with their environment in order to receive relevant information. However, the extent to which embodied experience is relevant to higher-level cognitive functions such as language processing in childhood has been little investigated. In particular, it is unclear to what extent the sensorimotor system is relevant for the processing of abstract language in children. Some studies support the notion that mental simulation, as well as connections between language and motor planning, are already observable in children [27,28,29,30,31,32]. It seems to be around the tenth year of life that traces of motor system involvement in action language processing become detectable. When comparing children (7–12 years) suffering from a Developmental Coordination Disorder (a disorder that affects the motor system) with typically developing children of the same age, Mirabella and colleagues [33] found that only the latter show an interference effect in a semantic judgement task. The children with the coordination disorder made more errors and had longer decision times when the action verb and the movement used to indicate the decision had the same effector. According to the authors, this interference effect is caused by the close connection between language-related and motor-related cortical areas that contribute to action language processing [33,34]. Mounoud and colleagues [35], on the other hand, reported a facilitation effect in children due to motor priming. After watching a video showing an action, children were faster to name visually presented tools congruent to that action. This facilitation effect correlated with the age of the participants. While the authors observed strong facilitation in 9-year-olds, the effect was weaker in eleven-year-olds and young adults [35].

To explore motor simulation in much younger children (preliterate, 3;5-5;5 years), Fecica and O’Neill [29] analysed the comprehension of movements in stories by manipulating the protagonist’s motivation to move. The children pressed a button to receive the next sentence. The speed of the button press correlated with the eagerness of the protagonist to perform the action described in the sentence, e.g., going to the dentist (longer reaction time) or going to get ice cream (shorter reaction time). The authors interpret the reaction time differences as a sign of strong content simulation. Moreover, they hypothesize that the active simulation of the story content supports children’s language understanding. Results by Glenberg and colleagues [31] are in line with these effects. Year one and year two pupils role-played a story with toys or imagined the stories’ content after reading it, whereas the control group simply read the story [31]. In the first two conditions, children showed better story comprehension compared to the reading-only condition. Indeed, by the age of seven, children possess the ability to create perceptual simulations of read or heard sentences within 1000 milliseconds [28]. Besides mental simulation, the Body–Object Interaction effect (BOI) is another argument in favour of sensorimotor activation in children’s language processing. The BOI can be scored in a word naming task by measuring verbal onset latencies and indicates how easily the child can interact with a word’s referent. Representations of words with a high BOI that are closely related to the body, such as *bicycle* (compared to a low BOI in *cloud*), are thus connected to sensorimotor experience [32]. By the age of eight, children perform as well as adults, whereas children younger than eight show no access to BOI-dependent sensorimotor information in reading. Adults and children above eight perform similarly, which the authors interpret as a sign for stronger semantic activation and neuronal feedback in sensorimotor areas. Moreover, James and Maouene [36] scanned children between four and six years of age while they listened to verbs and adjectives in an MRI scanner. They were able to show that listening to verbs activated cortical motor regions, whereas listening to adjectives did not. Not only was this activation detectable in young children, but it was partly effector-specific (activating hand or leg regions).

A little-studied question is the extent to which girls and boys differ in the development of embodiment of language. For example, studies often either show no gender differences in the processing of language between the ages of 12 and 23 [20] or do not differentiate their data by gender [23,36]. Gender differences that can be associated with embodiment of language are found only very sporadically, and effect sizes are comparatively small e.g., [37]. For example, males are more accurate in the areas of abstraction and mental flexibility, whereas females are more accurate and slower in the areas of attention and working memory. Overall, it appears that females reach a plateau earlier in some cognitive abilities such as motor speed, nonverbal reasoning accuracy, and speed [37]. Hauf et al. [38] found an unpredicted gender effect: they showed that overall, males responded faster than females in mental perceptual simulation during sentence comprehension, but found no difference in mental simulation itself. Only scattered evidence suggests that cognitive processing may differ between boys and girls in the age group of the present study [39,40]. Corthals [39] found that boys (ages 9–12) had slight advantages in metalinguistic awareness of homonymy, likely due to differences in maturation. There are also significant gender differences in metaphor use at later stages (mean age: 19.4 years) [40]. Brain maturation processes from childhood through adolescence and into early adulthood appear to be partially gender-specific and may differ between males and females [26,41,42,43]. These differences in brain maturation, including the maturation of language-relevant areas, raise the question of whether or not these differences also influence motor priming and language embodiment in the 11-year-old girls and boys of this study.

Given the very sparse data on gender differences in embodied language processing, we had not anticipated these in our original experimental hypotheses. Interestingly, however, when we conducted the experiment, we noticed a difference between female and male participants, in the sense that girls took longer to think about the language stimuli than boys did to respond correctly. This prompted us to add another research hypothesis related to gender differences in the development of embodiment of language after formulating the original hypotheses.

To summarise, these findings argue in favour of sensorimotor activation during action-related language processing in children. However, it is not clear whether this sensorimotor activation is also developed for abstract language at an earlier age. In the context of embodied cognition theories, the question of developmental influences on the processing of action verbs in abstract context and the potential activation of sensorimotor areas that are activated by motor priming has not yet been investigated. It is therefore unclear whether motor priming supports children in processing concrete as well as abstract language as it does in adults and whether there is a gender-specific effect.

To answer these questions, we conducted a behavioural decision time study in which eleven-year-olds mirrored movements and afterwards judged the semantic plausibility of sentences containing an action verb in either a concrete or abstract context. Using a comparable design, Schaller et al. [18] have already shown that self-performed movements led adults to make faster decisions when processing action verbs in both concrete and abstract contexts. These results argue for the involvement of motor areas in the semantic judgment of sentences. Based on these results, the aim of the current study was to find out whether children also profit from this motor priming as adults do. Therefore, we aimed to answer the following research questions:

- Do children show comparable motor priming to adults in processing action verbs in concrete and abstract contexts? Is the embodiment of language already similarly mature in them?
- Are there general differences between concrete and abstract sentences, as suggested by the well-known concreteness effect?
- Do children show different sensorimotor processing of action verbs in concrete and abstract context depending on their gender?

Accordingly, the following hypotheses were examined:

(1) After mirroring movements, children aged 10–12 show the same motor priming effects as adults when processing action verbs in concrete contexts. They decide more quickly about the semantic plausibility of sentences if they mirrored a movement before processing these sentence types than if they just sit still. In addition, they make fewer errors in the decision. Just like adults, children process concrete sentences faster in principle and make fewer mistakes.
(2) In contrast, when processing action verbs in abstract sentences, they show reduced or no motor priming, since the relationship between the semantic system for abstract language and the sensorimotor system may not be yet fully developed.
(3) Since girls behaved differently during the experiment in terms of mastering the language tasks, we assume that gender is a possible confounding variable and therefore include it in our evaluation.

## 2. Materials and Methods

### 2.1. Participants

In cooperation with a public school in Cologne (Germany), 65 pupils, who were around 11 years old (35 females, 30 males; 10.8 years, SD ± 0.5), were tested in the current study. We chose children of that age because at this stage, children have already reached a high proficiency in their mother tongue, but do still not perform as well as adults [22,24]. The parents or the legal guardians were informed and asked for a written permission to perform the experiment and use the data for scientific purposes. At no time point could the data acquired be associated with a participant’s name, as participant numbers were used for data collection and analysis. The school management and the class teachers involved were also informed and their consent was obtained. The children participated voluntarily. According to a modified version of the Edinburgh Handedness Inventory [44], all children were right-handed (laterality quotient 74.6, SD ± 21.9) and had German as their mother tongue. Since not all children could participate in the experiment for various reasons, we distributed toys as a reward to the entire class and not to the individual participating children.

### 2.2. Stimuli

**Figure 1:**
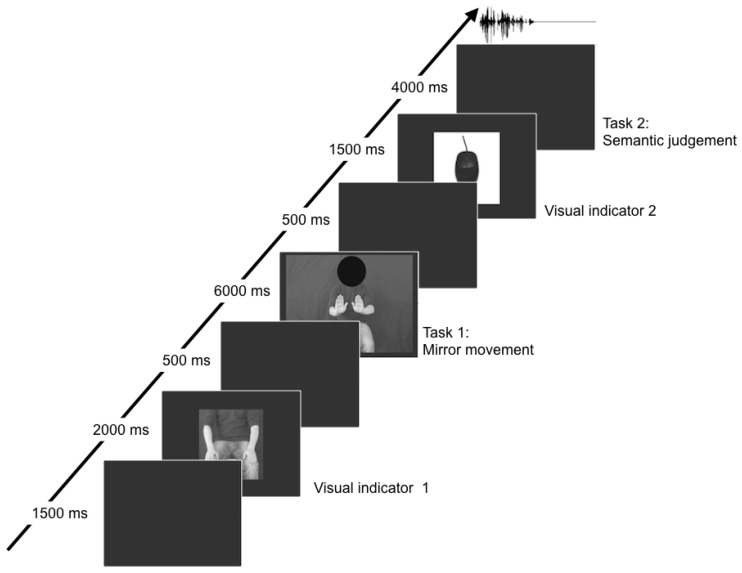
Duration and succession of events of a trial in milliseconds. Each trial started with a grey screen, followed by a picture displaying the starting position as instruction (visual indicator 1). Afterwards, a video was presented. In the movement condition, participants mirrored the movement presented in the video (task 1) and ended in the starting position with resting hands on their knees. In the no-movement condition, participants mirrored the video by staying in the starting position and sitting motionlessly. Next, a picture of a computer mouse signalled the participants to put their hand on the mouse (visual indicator 2). Subjects then listened to an auditory sentence and judged its plausibility via button press (task 2) (from [18], modified).

The experiment consisted of 48 trials with a duration of 16 s each. Each trial comprised a visual indicator (Visual indicator 1: freeze frame of a man with hands resting on his knees, Figure 1) followed by a short video (Task 1: mirror movement, Figure 1). A second visual indicator (visual indicator 2: picture of a computer mouse, Figure 1) was then presented, followed by an auditory sentence (Task 2: semantic judgment). Each sentence contained an action verb. After listening to the sentence, participants had to make a decision regarding the sentence’s plausibility by pressing a button.

Each trial contained a video lasting six seconds (Figure 1). The videos showed a male sitting in front of a grey background with his hands resting on his knees. His face was technically blackened with the help of a video editing program. Half of the videos showed a movement prototypical for the action verb in the successive sentence, while the other half did not show any movement.

Prototypical movements were selected based on the results of a former behavioural study in which participants were asked to perform movements related to auditorily presented verbs. These movements were filmed [45] and evaluated in order to identify prototypical movements for specific action verbs. The resulting video data for each action verb were analysed using five parameters to identify prototypical movements [18]. The parameters were:

- The type of movement;
- The direction of the movement related to the body position;
- The angle of the movement in a two-dimensional space;
- The speed of the movement; and
- The number of hands used.

The resulting prototypical movements were then performed by an actor, filmed, and used in the current experiment (see [18], for more information on the evaluation of prototypical movements). By choosing movements that were very specific and prototypical for each action verb, we expected a pre-activation of relevant motoric regions and thus a faster semantic processing of sentences.

In addition to the visual stimuli, each trial also contained an auditory sentence with an average duration of 1548 ms (SD ± 170 ms). In each sentence, an action verb was presented either in a concrete context (“Ich habe den Wagen gezogen.”/“I have pulled the wagon.”) or in a metaphorically used, abstract context (“Ich habe die Konsequenz gezogen. “/“I have drawn the consequence.”). Sentences with action verbs in concrete contexts had an average duration of 1524 ms (SD ± 136 ms), while the abstract action sentences had an average duration of 1570 ms (SD ± 201 ms). The duration of both sentence types did not differ significantly (*t* (96) = −1.345; *p* = 0.182).

The sentences were recorded with a semi-professional speaker. In a pre-test, the concreteness and the plausibility of the sentences were rated by participants who did not participate in the current decision time experiment (see [18]). Examples of the presented sentences are given in Table 1. Sentences with action verbs in concrete contexts represented an active movement, whereas action verbs in an abstract context conveyed a metaphorical meaning.

The stimuli were combined in a 2 × 2 repeated measures design. The two movement types (movement vs. no movement) were combined with the two types of sentences (concrete vs. abstract) (Table 1). Note that each action verb was presented in both a concrete and an abstract context.

In addition to the 48 experimental trials, the experiment also included 13 distractor trials. We selected those distractors from the pool of distractors in the adult experiment [18] that were maximally implausible semantically, to ease the task for the eleven-year-olds. We also ensured that none of the distractor trials were similar to the experimental trials. In the end, we had 13 distractor trials that were ideal for the current experiment. Their structure was identical to that of the experimental trials, and they also comprised videos both with and without movement. In contrast to the experimental trials, videos in the distractor trials showed unrelated movements and the sentences were semantically implausible (example: “Ich habe den Ausgang gegessen.”/“*I have eaten the exit.*”). Sentences and visual stimuli were taken from Schaller and colleagues [18].

### 2.3. Procedure

Five fifth grade classes were informed about the experiment in class. All children in a school class were tested on their handedness with a modified version of the Edinburgh Handedness Inventory [44] and were instructed by their class teachers and the experimenter. Only children who were interested in participating and could provide a written informed consent form by their parents had the opportunity to join the study. Before the start of the experiment, participants were asked for biographical background information such as their age, mother tongue, and knowledge of further languages (children whose first language was not German did not participate in the experiment; four children had contact with another language in their family but considered German as their native language and were not bilingual in a strict sense; one child was excluded because of possible bilingualism, see data analysis). An empty conference room in the school was prepared for the experimental session. Children were seated facing a computer screen against a neutral background with their backs towards the room. The experimenter made sure that participants had enough space to fulfil the mirroring task during the experimental session. The children were given oral instructions for the experiment to guarantee for a proper understanding of the task. They were informed that they would see short videos that either contained a movement that they were to imitate, or they were to imitate the behaviour of the person in the video by remaining motionless. Then, we told the children that they would hear sentences. We asked the children to listen to them and quickly decide by mouse click whether they were correct German sentences or not. They were not to think about this decision for a long time, but to act as instinctively as possible. After the children made clear that they had understood the instructions, they completed a training session to practice the experiment. Children were informed about the duration of the experiment before they started. Each child saw 48 experimental and 13 distractor trials, resulting in a total duration of 18.9 min.

The experiment was presented via a customised presentation software (“Sculptor”) running under Ubuntu (version 8.04.2). The participants were instructed to first mirror the posture as seen in the freeze frame presented at the beginning of each trial (hands on knees, Figure 1). After this, they watched the video and were asked to either mirror the perceived movement or to sit still (video without movement). None of the participants was confronted with the same combination twice. Subsequently, the children put their right hand on the mouse as soon as the freeze image of the computer mouse was displayed (Figure 1). They, then listened to the sentence and were asked to give feedback concerning the plausibility of the presented sentence via button press. Children pressed the right mouse button with their middle finger and the left mouse button with their index finger. The assignment of Yes and No responses to the mouse buttons was balanced across participants. The expected semantic plausibility judgement on the experimental trials was a press of the Yes-button, whereas the distractor trials were expected to be judged as incorrect and therefore received a No-button press. Decision times and accuracy were measured and analysed.

## 3. Data Analysis

Four children were excluded from the data analysis due to a very high number of errors (exceeding ± 2 standard deviations from the mean error rate across participants and low reliability values in the Cronbach’s alpha). Errors in this specific experimental setting are false alarms, meaning that participants rejected semantically plausible German sentences with action verbs in concrete and abstract context as semantically implausible (see Table 1). One child was excluded because of inconsistencies concerning the mother tongue (German was probably acquired as a second language). Thus, results from 60 children were included (34 females, 26 males; 10.8 years, SD ± 0.5, 10–12 years). Twelve trials were excluded in the data analyses because the number of errors concerning the plausibility judgment of these sentences exceeded two standard deviations of the mean error rate across sentences and low reliability values in the Cronbach’s alpha. Schaller and colleagues [18] created 96 trials for their adult experiment. Out of this pool of trials, each child in the current study completed 48 trials in order to shorten the duration of the experiment for the children. The combination of 48 trials taken from the pool of 96 trials differed between the children. Thus, each child did not complete the same 48 trials. For this reason, we excluded 12 out of 96 (not 48) trials. Decision times for correct answers that exceeded two standard deviations measured from verb onset were excluded as extreme values in each condition (−2 SD: 4 cases; +2 SD: 75 cases). After the elimination of extreme values, the analysis was based on 1562 decision time points. In the data analysis, decision times were calculated and compared by condition in a 2 × 2 repeated measures ANOVA (SPSS Vers. 23, IBM). The movement type (movement vs. no movement) and the type of sentence (concrete vs. abstract) were defined as repeated measures factors.

Because we discovered a possible influence of gender on language processing only during the experiment and had not included this variable a priori in our hypotheses, we could only include gender as a confounding variable in our repeated measures ANOVA.

Comparable analyses were carried out for the mean error rate. The wrong answers were calculated and compared by condition in a 2 × 2 repeated measures ANOVA (SPSS Vers. 23, IBM, Armonk, USA) with the movement type (movement vs. no movement) and type of sentence (concrete vs. abstract) as repeated measures factors and gender as covariate. Tukey tests were used for post-hoc comparisons.

## 4. Results

Below are the results concerning the decision times and the number of errors collected from the 60 children. Regarding the decision times, the 2 × 2 repeated measures ANOVA with gender as covariate showed neither a main effect of the sentence type (concrete vs. abstract) (*F*(1, 58) = 1.268; *p* = 0.265), nor a main effect of the movement type (movement/no movement condition) (*F*(1, 58) = 1.661; *p* = 0.203). The results revealed an interaction between the two factors sentence and movement type (*F*(1, 58) = 6.211; *p* = 0.016) and a three-way interaction of sentence and movement type with participants’ gender (*F*(1, 58) = 6.900; *p* = 0.011, see Table 2).

**Figure 2:**
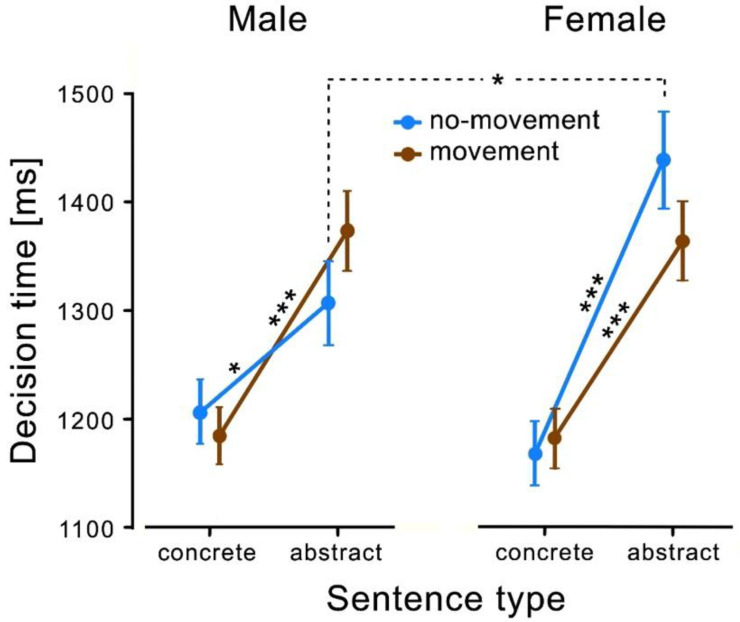
Decision times for all conditions separately for males and females. All children judged sentences with action verbs in concrete contexts significantly faster as plausible than in abstract contexts. In the abstract condition, males and females showed opposite effects. Males were significantly faster than females in the no-movement condition. Error bars display the standard errors of normalised data that are adjusted for repeated measures [46]. The results of the post-hoc Tukey tests are represented by asterisks (* = *p* < 0.05; *** = *p* < 0.001).

Post-hoc Tukey tests revealed a significantly faster decision time for concrete compared to abstract sentences in both the movement conditions and the no-movement conditions. Males (movement condition: *t*(25) = −4.990, *p* < 0.001; no-movement condition: *t*(25) = −2.470, *p* = 0.027) as well as females (movement condition: *t*(33) = −4.840, *p* < 0.001; no-movement condition (*t*(33) = −5.940, *p* < 0.001) decided significantly faster in the concrete than in the abstract condition. Furthermore, post-hoc Tukey tests revealed that male and female participants showed significant differences in decision times for the abstract sentences with no-movement only (*t*(58) = −2.170, *p* = 0.034), see Figure 2). Male participants were significantly faster in this condition than females. No significant differences could be found for the other three conditions.

Concerning the analysis of response errors, the 2 × 2 repeated measures ANOVA with gender as covariate showed a main effect of sentence (concrete vs. abstract) (*F*(1, 58) = 9.608; *p* = 0.003) (Table 3). Post-hoc Tukey tests revealed that children made significantly more errors when processing abstract sentences (*t*(58) = −16.600, *p* < 0.001). Additionally, there was a significant interaction between gender and sentence type (*F*(1, 58) = 4.538 *p* = 0.037) (Table 3).

Post-hoc Tukey tests revealed significantly more errors for abstract compared to concrete sentences in both the movement conditions and the no-movement conditions. Females (movement condition: *t*(33) = −8.040, *p* < 0.001; no-movement condition: *t*(33) = −10.460, *p* < 0.001) as well as males (movement condition: *t*(25) = −5.030, *p* < 0.001; no-movement condition (*t*(25) = −7.190, *p* < 0.001) made significantly more errors in the abstract than in the concrete condition.

**Figure 3:**
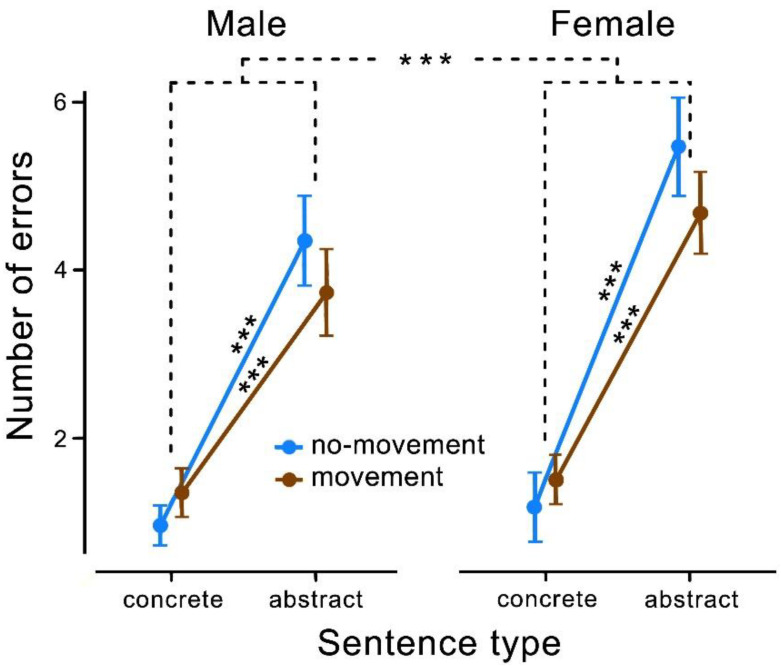
The error rate depends on the sentence type (concrete vs. abstract). Both males and females decided on the plausibility of sentences with action verbs in a concrete context significantly more often correctly than when verbs were presented in an abstract context. Females made significantly more errors in both sentence types. Error bars display the standard errors of normalised data that are adjusted for repeated measures [46]. The results of the post-hoc Tukey tests are represented by asterisks (*** = *p* < 0.001).

Furthermore, post-hoc Tukey tests showed that the female participants made significantly more errors than male participants (*t*(58) = −16.600, *p* < 0.001), which tended to be even more evident for both the abstract no-movement condition (*t*(58) = −1.860, *p* = 0.060) and movement condition (*t*(58) = −1.760, *p* = 0.080) (Figure 3). Overall, the error rate averaged around 22% for males (no-movement concrete: 8%, abstract 36%; movement concrete: 11%, abstract: 31%) and around 27% for females (no-movement concrete: 10%, abstract: 46%; movement concrete: 13%, abstract: 39%).

## 5. Discussion

The data of the current study revealed the following results: First, children did not show general motor priming comparable to adults when processing action verbs in concrete and abstract contexts. Second, children, like adults, showed an advantage of concrete over abstract sentences in both decision times and the number of errors. Third, however, concerning decision time, there was a three-way interaction between sentence type and movement type that differed for males and females. Whereas girls showed shorter decision times for processing action verbs in abstract sentences after a movement, boys showed shorter decision times for processing abstract sentences after sitting motionlessly. Fourth, with regard to the error rate when judging the plausibility of sentences, males made fewer errors overall than women in all conditions. The effect tended to be more pronounced for abstract sentences.

In contrast to findings in adults [18,19], children aged 10 to 12 did not show overall motor priming effects when processing action verbs. Only when processing action verbs in abstract sentences, where action verbs were used in a metaphorical sense, did an effect on motor priming occur, and it was gender-specific. Interestingly, only girls benefited from a previously self-performed movement when semantically processing these sentences. Boys, on the other hand, showed a negative priming effect by becoming slower in sentence processing when they had previously performed a movement.

According to embodiment theory, motor priming often facilitates the comprehension of action language, which is reflected in shorter reaction times. This is due to the pre-activation of sensorimotor areas that are also used during language processing, which has been shown in many different studies with adult participants [10,12,15,34,47,48,49,50]. Previous studies indicated that sensorimotor experiences may also play a role in children [29,30,51]. Although motor imagery processes are already detectable in children as young as five years of age, mental representations of movements continue to develop over the lifespan [52]. James and Maouene [36] showed that listening to verbs activates cortical motor regions such as the supplementary motor area. This activation can be demonstrated in children between the ages of four and six years and is partly effector-specific depending on the verb, i.e., different for verbs that designate hand or foot movements. Seven-year-olds already possess the ability to create perceptual simulations of read or heard sentences within 1000 ms [28]. A year later, at the age of eight, children access sensorimotor information and motor experience to process action language [32].

Contrary to our hypotheses, however, only girls benefited from a previously made movement, and only in semantic judgments of abstract sentences. An explanation for this finding must remain speculative at the present time but may be consistent with the study by Gur et al. [37], whose extensive analyses showed a weak gender effect in cognitive processes. They showed that men are more accurate in the areas of abstraction and mental flexibility, while women are more accurate and slower in the areas of attention and working memory [37]. Thus, it could be that boys in the present experiment are faster and less error-prone in processing abstract sentences than girls and do not benefit to the same extent from self-performed movement. Girls, on the other hand, are more accurate and slower at processing abstract sentences but benefit from self-performed movement. It could be that girls already have a different semantic system of abstract concepts and therefore, their brains were more attentive to connections between action verbs in abstract contexts and the movements that were made before making the decision. The assumption that girls thought more about the abstract sentences is also supported by their significantly higher error rate. With this interpretation, however, it remains unclear why no motor priming was found for the action verbs in the concrete sentences.

It is possible that, due to brain maturation processes at the age of eleven, under both conditions, children do not yet benefit from the movements, as is the case with adults [26]. The children’s language processing skills can still develop, which, among other things, depends on the continuous maturation of the grey matter from frontal to posterior cortical regions. At the age of 11.9 years, this reaches only 50% of the adult-like patterns [53,54]. The maturation processes going from childhood through adolescence up into early adulthood seem to be partly gender-specific and can differ between males and females [26,41,43,55]. For example, boys showed an average delay of 1–2 years in the maturation of the grey matter of the brain, namely around the age of 13 in the frontal lobe (girls around 11 years), in parietal regions around 10 (girls around 12 years), and in temporal regions around 17 years (girls around 16.5 years).

Another, simpler explanation could be due to the experimental setup. Children had to perform arm- and hand-related movements in order to improve the comprehension of arm- and hand-related actions verbs in concrete and abstract contexts. The button press used as plausibility marker, however, was also an arm- and hand-related movement, which may have interfered with the simultaneous processing of the action verb [56,57,58]. In this case, no influence of the movement can be seen, as the processing of some items may have been disturbed [14]. It has been shown that responding with a foot pedal improves motor priming when processing arm- and hand-related action verbs [57]. Another influence related to the experimental design could be the length of time between the end of movement and the beginning of the action verb, which averaged around 7000 ms and is much longer than in other studies on motor priming [14]. We used this longer time span because we assumed that children need more time to complete the task than adults. However, it could be that after this interval, a priming effect was possibly too weak to be recognised. In addition, in a typical ACE study, the processing of the action language is usually presented before a corresponding movement e.g., [12], while in this study, the movement took place first and then the language processing. This could also have had an effect on the quality of the priming [14]. There may have been an overlap between the motor priming and these experimental conditions. However, we chose the present experimental paradigm so as to better compare the results with the experiment previously carried out in adults [18]. It is possible that this experimental design is not appropriate for the study of language processing in children. These unresolved questions can only be answered in future experiments.

A further research question concerned the concreteness effect, which was very evident in the children as well as in numerous adult studies. Children at the age of eleven decided significantly faster on the semantic plausibility of concrete compared to abstract sentences and made significantly fewer errors when judging the concrete sentences. This finding is in accordance with results of former studies on the well-known concreteness effect that has been extensively studied in adult participants [59,60]. These results also agree with the results of Schaller et al. [18,19] on adult participants who performed the same paradigm and processed comparable stimuli. In addition to the faster processing of action verbs in concrete contexts, in our study, children at the age of eleven often rated abstract sentences as not semantically plausible, although they were semantically and grammatically correct German sentences. This higher number of errors hint at an incomplete mental representation of abstract concepts in children, which may have led to the described difficulties in understanding the meaning of action verbs in abstract contexts. This finding is in accordance with former research that described an incomplete mental representation of abstract concepts up to early adulthood [20,22,23]. By the age of twelve, children only have access to a basic definition of abstract concepts [20] and are unable to infer the figurative meanings of idioms [61]. This suggests that eleven-year-olds have not yet developed a complete mental representation of the abstract meaning of action verbs. Due to the fact that concrete language processing activates more distributed cortical networks than abstract language [62], action verbs in concrete contexts can possibly already be processed more effectively at the age of eleven.

Another finding concerns the interaction between gender and sentence. Here, it can be seen that female participants made more errors than males, both in evaluating concrete sentences and somewhat more pronouncedly in abstract sentences. Gur et al. [37] found that males were more accurate in the abstraction domains, and Corthals [39] found that boys (9–12 years) had slight advantages in metalinguistic awareness of homonymy. They made fewer errors in general, and specifically in the abstract condition, which fits well with our results. Corthals [39] suggests some reasons for such gender differences in language studies, e.g., preferences for the material presented or the study design. For example, male eleven-year-olds may have unintentionally benefited from the experimental design, which could explain why they made fewer errors [39]. However, due to the fact that a similar experiment was carried out in adults that showed no benefit for males, this argument seems rather unlikely. To resolve those questions, more studies are needed to directly evaluate these findings and provide clear indications for the possible gender differences at the age of eleven.

## 6. Conclusions

Taken together, the results suggest that children as young as eleven do not yet exhibit the same clear motor priming effects as adults. The embodiment of language does not appear to be as mature in these children, as evidenced by the fact that they often do not understand and misinterpret the meaning of action verbs, especially in abstract contexts. Thus, priming the motor system with their own movements helps children only to a very limited extent in the semantic processing of sentences. Here, however, an unexpected gender difference emerged. For girls, and only when processing action verbs in a metaphorical sense, a previously self-performed movement led to faster decision times, which speaks for an embodiment of language in them.

An explanation for these findings could be that female and male eleven-year-olds are still at different stages of development and are only in the process of completing the neural networks for their abstract concepts. The results of the current study argue for a weak theory of embodied cognition, according to which sensorimotor areas are involved in language processing to some degree, but are not yet as robust in children as in adults. Moreover, these developmental steps appear to differ in girls and boys. Therefore, further studies on children’s processing of action language at this age should be conducted, also to investigate possible gender influences.

A limitation of the present study is that the variable gender was not included a priori in the hypotheses. Another limitation is the experimental design, which was adapted from the adult version of a similar study [18] and may need to be better adapted to children in terms of the effects of timing, task order, and consideration of the influences of possible interference between the effector used for responses (hand movement) and the action verbs denoting the same effector (hand movements).

## Figures and Tables

**Table 1:** Examples of the stimuli (from [18]) used for the four conditions in the current experiment.

Condition	Movement Type	Sentence	Example Sentence
1	Movement: *pull*	Action verb in concrete context	“Ich habe den Wagen *gezogen*.” (*I have pulled the wagon*.)
2	No movement	Action verb in concrete context	
3	Movement: *pull*	Action verb in abstract context	“Ich habe die Konsequenz *gezogen*.” (*I have drawn the consequence*.)
4	No movement	Action verb in abstract context	

**Table 2:** Results of a 2 × 2 repeated measures ANOVA for the decision times (ms) with gender as a covariate. * = *p* < 0.05.

	F	df1	df2	Significance
Sentence (concrete vs. abstract)	1.268	1	58	0.265
Movement type (movement vs. no movement)	1.661	1	58	0.203
Sentence × movement type	6.211	1	58	0.016 *
Gender × sentence	3.559	1	58	0.064
Gender × movement type	1.922	1	58	0.171
Gender × sentence × movement type	6.856	1	58	0.011 *

**Table 3:** Results of a 2 × 2 repeated measures ANOVA for response errors with gender as covariate. * = *p* < 0.05.

	F	df1	df2	Significance
Sentence (concrete vs. abstract)	9.608	1	58	0.003 *
Movement type (movement vs. no movement)	0.000	1	58	0.994
Sentence × movement type	0.323	1	58	0.572
Gender × sentence	4.538	1	58	0.037 *
Gender × movement type	0.105	1	58	0.747
Gender × sentence × movement type	0.015	1	58	0.901

## Data Availability

The data presented in this study are available from the corresponding author upon request.
